# A case for a CUG-initiated coding sequence overlapping torovirus ORF1a and encoding a novel 30 kDa product

**DOI:** 10.1186/1743-422X-6-136

**Published:** 2009-09-08

**Authors:** Andrew E Firth, John F Atkins

**Affiliations:** 1 BioSciences Institute, University College Cork, Cork, Ireland; 2 Department of Human Genetics, University of Utah, Salt Lake City, UT 84112-5330, USA

## Abstract

The genus *Torovirus *(order *Nidovirales*) includes a number of species that infect livestock. These viruses have a linear positive-sense ssRNA genome of ~25-30 kb, encoding a large polyprotein that is expressed from the genomic RNA, and several additional proteins expressed from a nested set of 3'-coterminal subgenomic RNAs. In this brief report, we describe the bioinformatic discovery of a new, apparently coding, ORF that overlaps the 5' end of the polyprotein coding sequence, ORF1a, in the +2 reading frame. The new ORF has a strong coding signature and, in fact, is more conserved at the amino acid level than the overlapping region of ORF1a. We propose that the new ORF utilizes a non-AUG initiation codon - namely a conserved CUG codon in a strong Kozak context - upstream of the ORF1a AUG initiation codon, resulting in a novel 258 amino acid protein, dubbed '30K'.

## Findings

**Figure 1:**
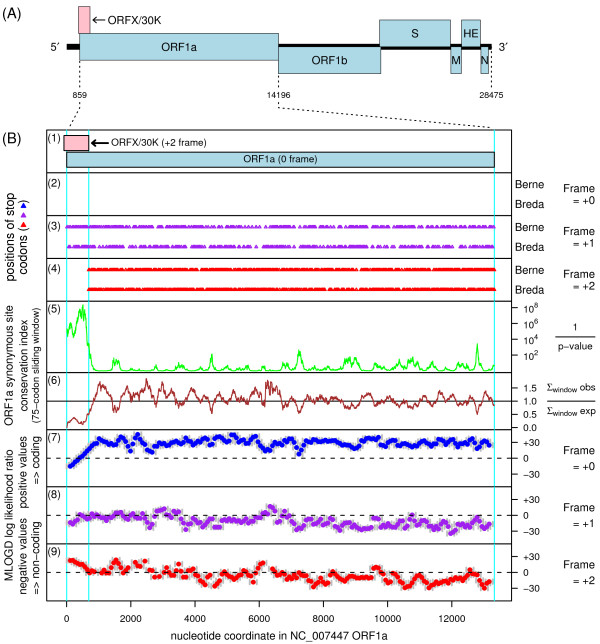
**Coding potential statistics for torovirus ORF1a and the overlapping ORFX**. **(A) **Torovirus genome map (Breda virus or Bovine torovirus [GenBank:NC_007447]; from [5]) showing the location of the proposed new coding sequence, ORFX. **(B1) **Map of the ORF1a region showing the proposed new coding sequence, ORFX, overlapping ORF1a in the +2 reading frame. **(B2-B4) **The positions of stop codons in each of the three forward reading frames. The +0 frame corresponds to ORF1a and is therefore devoid of stop codons. Note the conserved absence of stop codons in the +2 frame within the ORFX region. **(B5-B6) **Conservation at synonymous sites within ORF1a (see [11] for details). (B5) depicts the probability that the degree of conservation within a given window could be obtained under a null model of neutral evolution at synonymous sites, while (B6) depicts the absolute amount of conservation as represented by the ratio of the observed number of substitutions within a given window to the number expected under the null model. Note that the relatively large sliding window size (75 codons) - used here for improved statistical power - is responsible for the broad smoothing of the conservation scores at the 3' end of ORFX. **(B7-B9) **MLOGD sliding-window plots (window size 75 codons; step size 25 codons; see [8] for details). The null model, in each window, is that the sequence is non-coding, while the alternative model is that the sequence is coding in the given reading frame. Positive scores favour the alternative model and, as expected, in the +0 frame (B7) there is a strong coding signature throughout ORF1a *except *where ORF1a is overlapped by ORFX (see text). In the +1 and +2 frames (B8-B9), scores are generally negative, albeit with significant scatter into positive scores (a reflection of the limited amount of available input sequence data). Nonetheless the ORFX region is characterized by consecutive positively scoring windows in the +2 frame (B9). Note that, regardless of the sign (either positive or negative), the magnitude of MLOGD scores tends to be lower within the overlap region itself (B7-B9) due to there being fewer substitutions with which to discrimate the null model from the alternative model in this region of above-average nucleotide conservation.

The genus *Torovirus *belongs to the family *Coronaviridae *in the order *Nidovirales*. Species include Bovine torovirus, Equine torovirus and Porcine torovirus. As with other members of the order *Nidovirales*, these viruses have a linear positive-sense ssRNA genome encoding a large replicase polyprotein that is expressed from the genomic RNA (ORF1a and, via ribosomal frameshifting, an ORF1a-ORF1b fusion product), and a number of other proteins - including the structural proteins - which are translated from a nested set of 3'-coterminal sub-genomic RNAs (Figure 1A) [1-6].

Overlapping genes are common in RNA viruses where they serve as a mechanism to optimize the coding potential of compact genomes. However, annotation of overlapping genes can be difficult using conventional gene-finding software [7]. Recently we have been using a number of complementary approaches to systematically identify new overlapping genes in virus genomes [7-11]. When we applied these methods to the toroviruses, we found strong evidence for a new coding sequence - overlapping the 5'-terminal region of ORF1a (Figure 1). Here we describe the bioinformatic analyses.

Relatively little sequence data is available for the relevant 5'-terminal region of the torovirus genome. In fact there are only two non-identical sequences in GenBank (tblastn [12] of translated NC_007447 ORF1a; 2 Aug 2009) for the region of interest: [GenBank:NC_007447] - Breda virus or Bovine torovirus (derived from [GenBank:AY427798]) [5], and [GenBank:DQ310701] - Berne virus or Equine torovirus [4]. However these two viruses are reasonably divergent (mean nucleotide identity within ORF1a ~68%), thus providing robust statistics for comparative methods of gene prediction. The NC_007447 and DQ310701 ORF1a amino acid sequences were aligned with CLUSTALW [13] and back-translated to produce a nucleotide sequence alignment, which was analyzed with a number of techniques.

**Figure 2:**
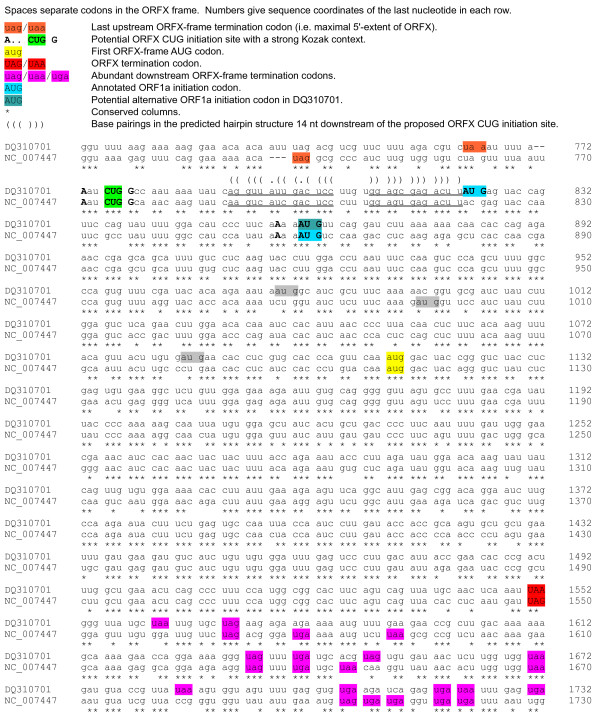
**Alignment extract showing ORFX and flanking regions**.

The first piece of evidence for an overlapping coding sequence is the presence of an unusually long open reading frame (229 codons; hereafter ORFX) at the 5' end of ORF1a but in the +2 reading frame relative to ORF1a (Figure 1B, panels 2-4). In fact ORF1a in Breda virus has 589 stop codons in the +2 frame (out of a total of 4444 codons), while Berne virus has 569 stop codons (out of 4568). In other words, approximately one in every eight codons in the +2 reading frame is a stop codon (see, for example, the last three alignment blocks in Figure 2). Thus the probability of obtaining an uninterupted 229-codon +2 frame ORF simply by chance is vanishingly small (*if *+2 frame stop codons within ORF1a are assumed to be randomly distributed, then the probability is of order *p *< 10^-10^). Moreover, there are 141 point nucleotide differences between Breda virus and Berne virus within ORFX, and yet the open reading frame is preserved in both viruses. The absence of stop codons may be linked to local nucleotide biases - indeed the mean nucleotide frequencies within ORFX (Breda virus) are A 28%, C 24%, G 20% and U 27% compared with A 27%, C 14%, G 23% and U 36% in the rest of ORF1a, so that the ORFX region is relatively C-rich and U-poor. However the simplest explanation for these nucleotide biases is simply the presence of an overlapping gene (i.e. ORFX) and the constraints imposed by having to code in multiple reading frames.

Next, the ORF1a alignment was analysed for conservation at synonymous sites, as described in [11] (but inspired by ref. [14]). The procedure takes into account whether synonymous site codons are 1-, 2-, 3-, 4- or 6-fold degenerate and the differing probabilities of transitions and transversions. There was a striking, and highly statistically significant (*p *< 10^-17 ^for the total conservation within ORFX), peak in ORF1a-frame synonymous site conservation at the 5' end of the alignment, corresponding precisely to the conserved open reading frame, ORFX (Figure 1B, panels 5-6). Peaks in synonymous sites conservation are generally indicative of functionally important overlapping elements, though such elements may be either coding or non-coding. In fact, high synonymous site conservation at the 5' end of long polyprotein-encoding sequences is a feature common to a number of RNA viruses and can not, in itself, be taken as evidence of an overlapping coding sequence. However the extent (229 codons) and degree (Figure 1B, panel 6) of the conservation here is unusual and, furthermore, the high conservation is not matched in the related coronaviruses. Thus an overlapping gene, viz. ORFX, provides the most obvious explanation for the high conservation seen here. (An alternative explanation is recombination, as in ref. [15]. However recombination does not provide an explanation for the other evidence presented in this report.)

Finally, we analysed the alignment with MLOGD - a gene-finding program which was designed specifically for identifying overlapping coding sequences, and which includes explicit models for sequence evolution in multiply-coding regions [7,8] (Figure 1B, panels 7-9). In contrast to the synonymous site conservation index above, MLOGD, when applied in the sliding window mode, does not depend on the degree of conservation *per se *(the sequence divergence parameter is fitted independently for each window). With just two input sequences, the MLOGD signal proved to be somewhat noisy (e.g. there are a number of positively scoring windows that clearly do not correspond to potential overlapping genes in, for example, the +2 frame; Figure 1B, panel 9). However the signal for ORFX was clear - with consecutive positively scoring windows throughout the ORFX region in the +2 frame - indicating, again, that ORFX is indeed a coding sequence. Moreover, the MLOGD score in the +2/ORFX frame within the ORFX region was significantly greater than the score in the +0/ORF1a frame, indicating that the ORFX product is subject to stronger functional constraints than the product of the overlapping region of ORF1a (which indeed has a negative MLOGD score towards the 5'-terminal half of the ORFX region). Consistently, further inspection showed that, in the region where ORFX and ORF1a overlap, ORFX has higher amino acid conservation than ORF1a (182/229 identities for ORFX, 153/229 identities for ORF1a).

In Breda virus (NC_007447), the annotated ORF1a AUG initiation codon is at nucleotide coordinates 859..861 and the first ORFX-frame AUG codon is at coordinates 1110..1112. However leaky scanning to this AUG codon is unlikely, due to intervening AUG codons in the ORF1a frame (1 in NC_007447, 3 in DQ310701; Figure 2). Instead we propose that ORFX initiation takes place at a CUG codon located upstream of the ORF1a AUG codon, at coordinates 774..776 (Figure 2). CUG is, apparently, the most commonly used non-AUG initiation codon in mammalian systems (reviewed in [16]), and this particular CUG codon is conserved, and has a strong Kozak context ('A' at -3, 'G' at +4; [17]), in both Breda and Berne viruses. The downstream sequence is predicted to fold into a hairpin structure that is identical between Breda and Berne viruses - despite a number of base variations - and that is separated from the CUG codon by 13 nt (Figure 2). Such structures - particularly at this spacing - have been shown to greatly enhance initiation at non-AUG codons [18]. Moreover, inspection of the sequence alignment upstream of the ORF1a initiation site shows that the majority (14/18) of base variations occur in the 3rd nucleotide positions of ORFX-frame codons, indicative of an ORFX-frame coding sequence (Figure 2). This pattern of base variation continues right up to the proposed CUG initiation codon. Initiation at a site further upstream is precluded by ORFX-frame termination codons and, consistently, the sequence further upstream does not maintain the reading frame and base variations no longer favour the 3rd position (Figure 2).

**Figure 3:**
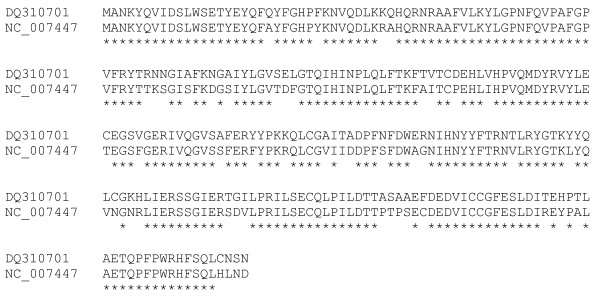
**Amino acid alignment for '30K', the translated ORFX**. Note, here the proposed CUG initiation codon is assumed to be translated by initiator Met-tRNA - resulting in an N-terminal methionine rather than leucine.

Initiation at the upstream CUG codon would give ORFX the nucleotide coordinates 774..1547 in NC_007447 and 776..1549 in DQ310701, resulting in a 258 amino acid product with a molecular mass of 30 kDa which, for want of a better designation, we tentatively name '30K'. The full predicted amino acid sequences are shown in Figure 3. Note that the product has only one methionine residue, making detection with [^35^S]Met difficult. Application of blastp [12] to the amino acid sequences revealed no similar sequences in GenBank (3 Aug 2009) - as expected for a gene created *de novo *via out-of-frame 'overprinting' of a preexisting gene [19,20]. Similarly, application of InterProScan [21] also returned no hits (protein motifs, domains etc).

It is expected that a large proportion of ribosomes should scan past the CUG codon and initiate at the ORF1a AUG codon - thus allowing synthesis of the replicase polyprotein - though the additional possibility that the CUG-initiation efficiency may be temporally regulated as part of the virus lifecycle can not currently be discounted [16,22].

Overlapping genes are difficult to identify and are often overlooked. However, it is important to be aware of such genes as early as possible in order to avoid confusion (otherwise functions of the overlapping gene may be wrongly ascribed to the gene they overlap), and also so that the functions of the overlapping gene may be investigated in their own right. We hope that presentation of this bioinformatic analysis will help fullfil these goals. Initial verification of ORFX product could be by means of immunoblotting with ORFX-specific antibodies, bearing in mind, however, that it may be expressed at relatively low levels.

## Competing interests

The authors declare that they have no competing interests.

## Authors' contributions

AEF carried out the bioinformatic analysis and wrote the manuscript. Both authors edited and approved the final manuscript.
